# Medical Deontology; or, the Duties and Rights of Medical Practitioners

**Published:** 1846-04

**Authors:** 


### Art. IX.

*Déontologie Médicale, ou des Devoirs et des Droits des Médecins dans l'Etat actuel de la Civilisation.* Par le Docteur Max. Simon.—*Paris,* 1845.

*Medical Deontology; or, the Duties and Rights of Medical Practitioners.* By Dr. Maximilian Simon. 8vo, pp. 590.

Medical ethics have never attracted so much attention from laymen as medical theology. The opinions of physicians on questions of faith have generally been held to be of doubtful orthodoxy; their morals have usually been found unimpeachable. Trustworthy as men, they have absurdly enough been deemed irreligious. In this respect, however, they have but endured the common fate of all philosophers. From the earliest period the student of nature has been thought little better than an infidel; and, doubtless, this popular belief in one sense has always been well founded. How is it possible to imagine that Anaxagoras, Epicurus, Hippocrates, Socrates, or other like-minded men, ever expressed an earnest faith in the mythology of their age? Or can it be supposed that the modern geologists (holding the cosmogonical opinions they avow) participate in the popular belief as to the creation of the world? The Dean of York, although wrong alike in his theology and his philosophy, is true to the instincts of the ignorant multitude whose sentiments he expresses. Philosophy will ever war with mere dogmas, and medical science is philosophy *par excellence.*

And yet we venture to assert that there is not a more truly and sincerely pious, nor a more earnestly moral class of men than our own much calumniated profession. It ill becomes any profession to laud and magnify itself; but the avowal and defence of truth should not and shall not be sacrificed to the paltry fear of being thought vainglorious; or of being considered as emulous of those canting knaves who use religion as a cloak for pride, ignorance, or avarice. The cultivators of medicine, equally at least with the members of any other profession, may, without usurpation, to use the words of Sir Thomas Browne, assume the honorable state of a Christian, and we dare assert that none disgrace that honorable name less than medical practitioners.

If proofs of these assertions were wanting, they could not be more amply or aptly supplied than by the book before us. We subjoin an example, and we do this with the most perfect confidence that none but the most narrow-minded bigots can fail to recognize in it the pith and essence of Christ's teaching,—the marrow of that new law of mutual love which he left to mankind.

"With the man who is responsible to God alone for his decisions and actions regarding a thing so precious as human life, the thought of God should be ever present. A philosophy through which the sap of this fruitful thought does not circulate will be powerless to guide the physician at all times in the midst of the numerous quicksands he must meet with in the exercise of his profession. Medicine may be traced to God through the sympathy which the appearance of suffering awakens in us; but as a science of so high origin it only completes its work by asking from charity its love and its devotion. The physician who takes the light of this dignified philosophy as the guide of his conscience may fail, but his fault will only be imputed to the imperfections of science. Understanding the dignity of human nature and the profound objects of life, he will devote himself entirely to the study of a science which can influence so decisively the individual destiny of men. Prudent and circumspect, he will not be seen to adopt hastily those unripe theories which sometimes pass over a generation like a destructive epidemic. In those cases, in which both theory and experience refuse their teaching, he will adopt the method of a prudent expectant treatment. To whatever rank of society he may belong, the sufferer will be his brother by the double relationship of pain and hope. Beneath the most hideous rags of misery he will recognize the indelible marks of the sufferer's heavenly origin, and with tender solicitude heap upon him the most devoted attentions. If in practice he meets with one of those difficult cases in which one means only can save the patient, and the use of those means may destroy his practice, if it fail, he will know how to do his duty, and endanger his reputation and success. Good and kind to all, he will not use his amenity of language as a privilege to gain wealth or rank. He will be aware that suffering spiritualizes a man, if the expression may be permitted, and gives for the moment to the most unpolished natures a delicacy and sensibility which rudeness of manner deeply hurts. He will be as affable and agreeable at the truckle-bed of the poor as in the perfumed boudoir of the rich; and thus both fulfil a rigorous duty and secure the action of those curative agents which science may direct.

"But conscience abandoned to her own guidance may stumble in the dark paths along which she might lead us: she is accessible to passion, and is led by fantasies, as is every power not attached to something fixed and immovable. It is necessary to mount higher and find a surer guide; it is necessary to ascend to that Christianity itself which affords infallible instructions suited to every station of life—to that Christianity which, expressing its doctrines in the one word CHARITY, is wonderfully allied to a science of which the essential object is the solace of human suffering. The men who have left behind them the most glorious names in the history of our science, Bayle, Van Helmont, Stahl, Sydenham, Boerhaave, Hoffman, Van Swieten, Winslow, Esquirol, &c., have well understood that it is in Christianity only the physician should seek that light and strength of which he has need, to maintain him equal to his difficult mission." (Introduction, p. 24.)

M. Simon at the outset, thus takes the sublime morals of Christianity as the fundamental principles of his medical ethics. Christian morals are beyond all controversy, whatever may be thought of Christian dogmas.

DEONTOLOGY is a word adopted from Bentham, who used it to signify morals, or the science of duties. M. Simon uses it in the double sense of duties and rights, observing that the true meaning of the root of the word, δέον from δεῖ, oportet, warrants this double use of the term. Doubtless all duties are reciprocal; the rights of medical practitioners are the duties that society and individuals owe to them, as their duties to society and individuals are the dues or rights of the latter. A general view of these duties and rights is given in a lengthened introduction; it is a recapitulation, in fact, of the contents of the work.

M. Simon divides his subject into four books. The first treats of the duties of practitioners to themselves and to science; the second of their duties to the sick; the third of their duties to society; and the fourth of their rights, or, in other words, the duties of society and of the sick to them.

In treating the subject of the first book, he first inquires what motives ought to guide a practitioner in the cultivation and practice of his profession? Our author well observes that the liberal professions sink lower and lower in public estimation. Medical practitioners, in particular, are full of complaint of the little honour and regard shown them by society. We should, however, be inclined to doubt whether the present generation croak more or in a more melancholy strain than the last; or whether the profession is really in less esteem than formerly. M. Simon believes this decadence to be real, and thinks the reason thereof is manifest enough. Few practitioners are guided by the principle of action which can alone secure them the regard and esteem of society. They are not devoted to the common interests, but to their own. They live *out of* society, and not *for* society; so society owes them nothing, except a watchful guardianship that they shall not under the sacred garb of the profession act the medical Tartuffe, and destroy rather than restore. There is nothing new under the sun; men are always equally selfish; the object of desire may differ, their greediness is ever the same.

Not long ago we happened to turn over several volumes of the earlier medical journals, and we were struck with the identity between the croaker of the existing and the last generation. In the earlier years of the present century medical reform was vigorously agitated by Sir Joseph Bankes, Dr. Beddoes, Dr. Harrison, and others. Much discussion was excited, the colleges were disturbed from their slumbers, the ultima Thule of the profession aroused. And what was the great characteristic of the discussion? Utter, barefaced selfishness. The London College of Physicians sent advertisements to the political journals, denouncing Scotch graduates as illegal practitioners; the King and Queen's Colleges of Physicians of Ireland answered the call for reformation by complaints regarding the encroachments of surgeons and apothecaries, and by a demand for further monopoly and more restrictive powers. Then as to the general practitioners, Dr. Beddoes, in his commonplace book (date 1808) observes, "the quantities of medicine ordered drive people to quack medicines. People can drop the latter when they please, but *verecundia erga medicum* makes them keep on longer with the former than either their stomach or their purse can well endure. To rescue the public from the stomach part of this evil, it will be proper to enact that the apothecary shall charge for more draughts than he supplies, as used to be the case with post-horses in France." In 1803, a country surgeon writes to a journal, "It is a melancholy consideration that whilst almost every class of men are in a state of improvement, the Faculty are certainly going retrograde; I speak to their revenue; competition has reduced the profession to the lowest ebb, consistent with comfortable support. . . . . . In short, such is the state of the country practitioners, that empiricism must soon supersede science, if every facility is not afforded him to procure information!" Complaints were loudly uttered not long ago against the *drenching system;* but, bad as it still is, we are sure that never did it flourish less than at this moment.

There is more hope, then, of a moral regeneration of the profession than croakers will allow; and there is the more hope, because the selfish are beginning to discover that a thorough devotion to professional science and duty is the surest, if not the shortest, way to wealth and esteem. They even doubt the wisdom of those public advisers who speak of the incomes of quacks as so much taken *from* the pockets of regular practitioners. Although we think M. Simon too enthusiastic, we would not despise the high moral feeling which demands this devotion for the sake of virtue and for the love of God. But the incentive of a personal advantage to be obtained is a true and right incentive. How does the Wise Man incite to the love and pursuit of wisdom or virtue? By describing the advantages she can confer. "Length of days are in her right hand: in her left hand are riches and honour." Our author's sentiments are as follow:

"Follow the physician in imagination through all the situations in which the requirements of his laborious profession place him; follow him to the palace of the rich, or to the hut of the pauper; among the poor artisans who want air, or the peasants who have that alone; follow him amid the epidemics which decimate the populations around him; amid the contagion whose deadly miasm he may absorb at every pore; to the courts of justice, where his scientific words may advance or injure the most important interests of society, or of the individual; in short, follow him in the numerous directions where his office necessarily calls him, and you will always find him surrounded by the most imperious obligations. Now, the abstract idea of duty and the absolute principle of moral obligation are alone able to maintain him constantly in strength for his duties. He only who directs his conduct by this higher motive will make art equal to science, and know not the hesitations of devotedness which are nourished from sources less pure. The man who submits to this moral regimen contracts a habit of virtue—of self-denial—which will render his duty easy whenever it shall be opposed to his own numerous passions." (p. 53.)

The duties of medical instruction and authorship are to be regulated, our author thinks, by the same high principles of unselfish devotedness. The greater his influence, and the wider his sphere of action, the more incumbent it is upon the teacher to purify the sanctuary of his conscience, and generously expel every interested motive. In medical science false ideas and crude theories may easily develop a dangerous method of treatment. Vanity, pride, avarice should be so chastened as not to obscure and deform the scientific page. M. Simon might have referred to a tribe of crawling scribblers, whose vocation is the pursuit of a miserable notoriety by the publication of unmitigated falsehoods. These men report cases they never witnessed, assert cures they never performed, record facts that never occurred. We promise ourselves some day the pleasure of impaling a few specimens of these creatures on our critical pens, and placing them with outstretched wings in our bibliographical museum.

What is the effect of medical studies and practice on the morals of the practitioner? M. Simon thinks they have a baneful effect. The pursuit of descriptive and pathological anatomy familiarizes the practitioner with man in a state of degradation. The majesty of death teaches him nothing, and affects him not; the corpse before him is a "subject," and nothing more. The infirmities of diseases and the nudities of death are necessarily ever present with him, and life only appears as a veil thrown over them. If the feelings thus excited are not counterbalanced by an abiding conviction of the dignity of human nature, the tender sympathy and the nobler instincts of humanity will be injuriously weakened, and with them the mental powers themselves. An impudent cynicism is excited, a looseness of language and deportment follows, and the student becomes the Don Juan of the dissecting-room.

The religious opinions of the practitioner suffer also, and he becomes affected with infidelity. On this point M. Simon dilates considerably. The charge of heterodoxy and atheism, as we formerly remarked, has been brought against philosophers, and especially physicians, from time immemorial. The "Beware of Philosophy" of the priest is much more ancient than the "*cave canem*" of the Romans. It is quite true that many philosophers (and medical practitioners are to be classed with them) hold opinions differing from the popular belief, and on this difference the charge of atheism has been ignorantly founded. Of those absurd dogmas which medical practitioners, especially in Catholic nations, are categorically required at once to believe and explain, Sir Thomas Browne, in his 'Religio Medici,' humorously remarks: "There are a bundle of curiosities, not only in philosophy, but in divinity, proposed and discussed by men of supposed abilities, which indeed are not worthy our vacant hours, much less our serious studies. Pieces only fit to be placed in Pantagruel's library, or bound up with Tartaretus de modo cacandi." The materialism of the medical practitioner is often no materialism at all, but only a dissent from the pagan doctrines that have crept in amongst the Christian. His habit of exact dissection; his knowledge (of the most demonstrable certainty) that the phenomena of mind, so far as we are able to understand them, are dependent upon organization; the constant proofs he has of a far more profound scheme of creation than the generality of divines teach, or than he can fathom; and the frequent illumination of that vast abyss of life which science has disclosed to him by her own lightning flashes, all tend to impress his mind with the greatness of his ignorance and the necessity of faith. Where fools rush in he fears to tread. "We are men, and we know not (scientifically,) how: there is something in us that can be without us, and will be after us: though it is strange that it hath no history."

The repeated witnessing of pain in our fellow-creatures has necessarily a tendency to blunt the sensibilities, and in so doing to render us less useful in administering solace. This is true in some degree, and ought to be combated by a sedulous kindness of manner; but much depends upon the natural character of the practitioner. Many are born with tender and compassionate hearts, and to such the repeated contemplation of suffering acts only as a stimulus to greater efforts. On the other hand, the medical practitioner or student is peculiarly liable to suffer from hypochondria. The study or practice of his profession effects the same change in his nervous system as is induced in the layman by the perusal of medical books. There, we suspect, are few practitioners of eminence who have not been consulted by students or young practitioners for some imaginary disease or other, but most frequently, perhaps, for supposed pulmonary phthisis, or disease of the heart.

M. Simon advocates the propriety of a greater respect for the dead than is usually exhibited by the profession, especially by students. We confess that we have not witnessed without horror the profane desecration of the human remains brought into the dissecting-room. We trust the prosectors of the present day have changed all that, and humanized the student. It is impossible to escape altogether from the benumbing influence on the sensibilities which familiarity with corpses develops, but it may be moderated and counteracted. One absurd means recommended for this purpose by M. Simon is, that the walls of the anatomical theatre and dissecting-room be covered with short philosophical sentences. In hospitals respect for the dead is really an imperative duty. The dying, in witnessing the treatment of the lifeless corpse, witness the mode in which their own will be shortly treated. They not only fear to die, but they fear any indignity to their remains. It is possibly a selfish superstition, but it is strong and universal. How many gorgeous tombs and monuments has it not raised? How often has the high-minded and brave Greek or Roman, touched by the poetic mythology of his age and nation, feared less to die than to remain unburied?

> "Hæc omnis, quam cernis inops inhumataque turba est;
> Nec ripas datur horrendas, nec rauca fluenta
> Transportare priùs, quam sedibus ossa quiêrunt."

M. Simon strongly objects, and we think with reason, to the sale of bodies practised at the French hospitals. In England, he observes, this disgraceful practice was carried on only by a few infamous men: in France society is an accomplice in the profanation, by tolerating and legalizing it.

M. Simon devotes a chapter to the consideration of the intellectual and moral qualities which the practitioners should possess or acquire. The more human life is valued, the more valued will the practitioner be. Independently, however, of any estimate placed on his services by society, feeling, as he must feel, how sacred a thing human life is, the conscientious practitioner will spare no labour so to qualify himself for his duties, that the assistance he may tender shall be the most effectual possible.

The first requisite is a continued mental activity—a habitual course of mental gymnastics. The practitioner should be *always* a student, not merely of the practice of medicine, but of those kindred sciences which develop the mental faculties, and give them harmony. This method will prevent him degrading his profession into a matter of mechanical routinism; will enable him to afford a just and not a short measure of skill to the patient who places his confidence in him; and will render the fulfilment of his onerous duties a pleasure, and not a task. This love of his art will further develop in him a love of his science, and thus he will be incited to those labours of investigation by which science can alone be advanced, and to the scrutiny of the facts and theories which continually come from the prolific laboratories of medical philosophers. However extensive his experience may be, he will still have need of the experience of others. The physiognomy of disease is so changeable that no nosography can accurately define its ever-varying phases; the causes of diseases are so numerous and novel, that no therapeutics can unravel their complexities, and eradicate or check them. The habit of close and accurate observation should be diligently acquired as the best means of developing that medical hyperæsthesia of the senses, termed tact. It has been much the custom to decry the skill and tact of scientific as opposed to empirical practitioners, and we suspect that this disparagement of the former has been founded upon the different circumstances of the two classes. While the one has used more assiduously the perceptive organs, the other has put the intellectual or reflective into greater exercise. The latter has indulged in the generalization of phenomena; the former in their analysis and relations. It is, therefore, of great importance to the scientific practitioner that he should seek after truths that may be applied rather than after abstract truths; that he should minutely investigate pathological phenomena day after day, either to illustrate known principles, or to discover unknown; that he should experiment less in the laboratory, and study more the experiments of nature. We will answer for the success of this method in forming a sound and successful practitioner. Nothing can compete with that union of the reflective and observing faculties which we have just recommended.

We have always thought a mature middle age the ripest period of life in a physician. To the young man we would say beware of precipitancy and self-confidence. The art is not so sure as the lecturers and professors lead you to imagine. Their systems are necessary for the development of their subject; they teach you what will cure rather than what will slay; the latter they leave you to discover yourselves—forgetting, perhaps, that your ardent imaginations differ widely from their mature experience. But if youth has its unbridled energies and undarkened hopes, age has its fears and cowedness. The one is really as dangerous as the other; it is as disqualifying to hope nothing from medical aid as to hope everything; we may kill by tardiness and inertness as effectually as by precipitancy and the so-called heroic treatment. Whether it be preferable to die *secundum artem*, or *secundum naturam*, may be a nice question, and, indeed, is perhaps only a matter of taste; most people prefer not to die at all. If a man be naturally of a timid temper he is scarcely fit for the duties of medical practice: decision and energy are as necessary at the bedside as on the field of battle. If such a man wish a sacred calling, let him put on the priestly gown. Even if by a strong effort of study he be deeply convinced of the mighty powers of medical art, still his natural temper will continually stay his hand while wielding those powers. The meditated blow will be checked! the act that will inflict present pain, but secure future ease, will be undetermined so soon as decided; the practitioner will be a vacillating doubter. But to the man who has from his organization, or his training, acute senses, quick perceptions, a comprehensive mind, steadiness of temper, firmness, and decision—to such an one the science and art of medicine must present a field for happy and contented employment that no other profession can offer.

It cannot be denied that the minds of some practitioners are imbued with an abiding scepticism as to the power of medicine as an art, and its reality as a science. When a contemplative and naturally doubtful mind glances through its history, it necessarily revolts at the numerous contradictions afforded. There is to be seen a continual conflict of the most opposite principles and modes of treatment. Now it is true the one theory or method is in the ascendant, but by the time it attains its climax it has called forth the antagonist that will surely supplant it, and rise to eminence in its turn to be again supplanted by another. And what is true of general doctrines is also true of special remedies. There is a mode or fashion in physic: a conventionalism is established, and men of medium powers lisp its phrases, thinking their wisdom oracular, until the ebbing tide leaves them ashore amidst the debris of their favorite methods and theories. It may be doubted, however, whether theories have much *permanent* influence on practitioners in general, and on the treatment of diseases. The therapeutics of the Hippocratic writings do not so widely differ from those of the present day as might be expected from the lapse of two thousand years; and the difference is still less obvious if the beginning of the last century be made the point of comparison. The animists, the intro-mathematicians, the Brownists, and the Broussaists, are but a little more lifeless than the "liver doctors" and the "heroes" of the present century. Certain principles and methods of treatment reappear or arise amidst the flux and reflux of medical opinions, however deeply they may have been overwhelmed. These must be true principles, having stood the test of time; and, consequently, scepticism is unjustifiable. The history of medicine should form part of the student's course; not a history of dates and names, but a history of evolution and development of doctrine—the embryology of medical art. This would be the best safeguard against the foolish conviction that there is no help in it.

M. Simon devotes a chapter to the literature of the profession, and takes occasion, in the first place, to impress on his readers the necessity of cultivating a good style. He objects to the dry, costive method of communicating facts; he would have the language elevated, noble, dignified, picturesquely elegant. Great facts must be described in great language, or they are overlooked, or appropriated by the more elegant writer. Many forms of disease can only be described in elaborate and forcible sentences; the style must be eminently descriptive. The scribblers of the profession are a true plague; they fill the journals with "cases," the truth of which is often questionable, and when not questionable are so imperfectly narrated as to be useless. The object aimed at by them is a short and valueless notoriety. This attained, these medical Bombastes care not how they fill up the columns of the weekly journal with their rubbish, for which the honest hard-working practitioner has to pay; or how they load the index through which the true litérateur has to wade. We certainly think the editors of weekly journals should exercise a stricter discrimination in the admission of communications, as well for their own sake as for the sake of their subscribers. Lies and lumber they should carefully eschew.

A good style can hardly be attained (except by the genius) without a good education; and genius itself often combats in vain with the defects arising from an imperfect literary education. The expression of great physical truths requires as much cultivation of the intellect as the expression of great moral truths—nay, we think more; for the former is addressed almost exclusively to refined minds, while the latter, being studied by every man, readily finds a welcome and an echo in every man's heart. This intellectual cultivation would develop more of sentiment and poesy in medical writings.

M. Simon justly animadverts on the absence of anything like sympathy for human suffering in the works of modern writers on pathology. The study and practice of pathological anatomy seem to have frozen up the springs of human feeling. Sufferings are described with as much icy coolness as the characteristics of a fossil reptile; or rather with more; for the geologist often warms with enthusiasm while he writes. He pictures the unwieldy creature in detail; he measures its teeth and jaws with undisguised interest; describes its tail, its claws, its eyes; he sees it in imagination living and gambling in its native haunts; now raising its long snout above the warm mud in which it is snugly ensconced, now cleaving with frightful velocity the tepid waters of its home. Even in our northern clime, he pictures skies above it of tropical splendour; flowers, and shrubs, and trees around it of tropical grandeur; birds and insects flitting near it of tropical gorgeousness. But the pathologist having recounted his case, it may be of hydrophobia, arachnitis, or tetanus, with a deliberate minuteness, winds up with a detail of the post-mortem examination, and a self-congratulation at being able to communicate particulars of such an "interesting case." There is not one word expressive of sympathy or kindness.

There can be no doubt that this cold though truly sciential style impresses the lay public more unfavorably, and is more injurious to the dignity and honour of the profession than many would willingly allow. M. Simon seeks not from the pathologist a funeral oration, or sepulchral poetry in the form of medical prose. He would present as models of a suitable style the Enchiridium of Hufeland, the Practical Medicine of P. Frank, the Pathology of J. Frank, or the Essays of our own Fothergill, especially that in which he describes pulmonary phthisis.

Chastity of language is another point M. Simon dwells on as necessary in medical literature. He thinks a writer on subjects which were formerly thought to demand the use of the Latin language should go direct to his subject, and use none but strictly technical words and phrases. Chlorosis should not be "fièvre d'amour;" the pubes is much better called pubes than the *mons veneris;* syphilis is the pox, and not the ladies' fever. Many of M. Simon's remarks on this head are much more applicable to French literature than to our own, if we except 'The Silent Friend,' 'C. . . . . on Marriage,' and similar bestialities of typography. For these, however, courts of justice are answerable, not the profession.

A chapter is devoted to truthfulness as a duty to science. In discussing this point, M. Simon severely animadverts upon those plagiarists, the pirates on the high seas of literature, who not only rob their neighbours, but disfigure and pervert to base uses their goods. The duty of truthful observation is strongly set forth; and especially in all cases the duty of showing the negative as well as affirmative side of every case or discussion.

The passions of medical men have a special notice, and first comes personal rivalry. No science lulls selfishness to rest—medical science as little as any. Satirists have always delighted to picture the jealousies and personalities of medical practitioners. A dialogue from Molière may be paralleled even now:—

"*M. P.* Je soutien que l'émétique tuéra.

"*M. des F.* Et moi, que la saignée la fera mourir.

"*M. P.* C'est bien à vous de faire l'habile homme!

"*M. des F.* Oui: c'est à moi; et je vous prêterai le collet en tout genre d'érudition.

"*M. P.* Souvenez-vous de l'homme que vous faites crever ces jours passés?

"*M. des E.* Souvenez-vous de la dame que vous avez envoyée en l'autre monde il y a trois jours?"

Nothing can be more destructive to the character and influence of the practitioner than scenes of this character. Yet the most polite, the most placid, and the most urbane may be so soured by the impudence and ignorance of some vulgar pretender, that, in spite of himself, he may be a principal actor in a scene like this. We believe it quite impossible to exclude men of low minds from the profession; the only remedy is for the high-minded to eschew them. An offence against good-breeding is sufficient ground for a professional excommunication. If the offender cannot be driven from the field, let him have it to himself. It is better in every way rather to flee than strive; the public ultimately judges right. The remedy for professional jealousies is frequent intercommunication. A good dinner once a quarter at the "Royal" or other hotel would heal the professional feuds of a large town. The man of science who practises his profession for the love of it may smile at the sensualness of the means, and truly it is not the remedy *he* would require; but most practitioners are men of the métier, and they like a trades-union, as well as any class. We wish there was a medical guild in every large town, with an ample dinner-fund; good fellowship, we are certain, would the more abound, and, with that, unity of purpose, honour, and public esteem.

Medical criticism is the next subject discussed by our author. He justly remarks that the practitioner who writes for the love of the truth, and to advance his art rather than himself, will willingly submit to fair criticism, and readily acknowledge errors. Prejudices and errors may be ridiculous only in many departments of science; in medicine they are murderous. Impartial, independent, and strict criticism is a duty incumbent on the medical critic; he cannot yield to personal animosity, or cowardly complaisance, without incurring a serious responsibility. As the vigilant sentinel of true science, he must stop alike the intruder and the foe, the hurtful and the useless, the fanatic and the selfish. We will boldly state, because we can honestly state, that such have been our critical canons. Often have we controverted views and facts, when approval would have been a pleasanter task; often have we applied the lash of our satire with regret that our pages should be devoted to the ignoble purpose of punishing the quack and pretender. Often have we desired to encourage the vigorous growths of science, while we endeavoured to repress the superabundant vegetation—rooting up weeds while we thinned the brushwood.

M. Simon refers with impartiality to the false patriotism of those critics who see no merit except in their countrymen's writings. The French and Italians are more remarkable than either the English or Germans for this narrow feeling of nationality. M. Simon refers specially to the reiterated attacks of the French philosophers upon Newton in favour of Descartes as a memorable example; but he thinks, on the other hand, that the discovery of Laennec has been treated with neglect in other countries;—the English and German physicians, he remarks, still contest its value. Surely M. Simon is little acquainted with English medical literature or practice, to make this assertion with respect to England. It may be true that neither Hufeland nor Frank have adopted the stethoscope, but the neglect of that valuable instrument by them should be rather attributed to the infirmities of age than the prejudices of country.

Literary comradeship, M. Simon thinks, has a baneful influence on fair criticism. The mutual admiration displayed in literary cliques is more ridiculous than dangerous; at least in England, where we have so many societies, sections, parties, and organs of parties. The repulsion of incongruous minds counterbalances the attraction of the congruous so soon as it is maudlin. If this were not so, there is, we believe, no better remedy for the spirit of cliqueism than an independent journal. The impudence of some men at professional reunions is astounding; first, in praising their superiors, and, secondly, in thinking their praises worth having.

What are the duties of the practitioner towards the sick? The first and the most imperious is, never to refuse medical aid to any one desiring assistance. And this he must do impartially, not having respect to persons. The only rule of preference should be that based on the degree of suffering. Nor is it by any means right that the practitioner should unequally regard the rich and poor, the good and the bad. Like the sun and rain of Providence, he must vivify and cherish both the just and unjust. We know that there are many puritanical persons who think themselves entitled to judge others, and who would relieve only the "respectable;" thus is their self-righteousness breaking one of the first rules of Christian charity. The true-hearted and really good practitioner will never allow such motives to influence him. Where shall inquiry into character begin?—where end? The distribution of medical relief, if made according to this rule, should be at least just, and the secret wickedness should be estimated even as weightily as the open. The undetected criminal is often the consummate hypocrite. The question scarcely, however, admits of serious argument.

The patient, M. Simon observes, whoever he may be, should be treated with unvarying kindness. In particular, his own history of his sufferings, even though wearying and prolix to the listener, should be heard patiently. The patient hearing is of itself no small solace to the sick man. Numerous engagements may not always permit a prolonged audience, especially when the patients are poor. *Il faut vivre.* Still, if the sick person be cut short in his, to him, touching history, it may be done so kindly that he willingly acquiesces. A kind word or two, a pleasantry adapted with perfect good taste to the man and the moment, a friendly glance, or pressure of the hand, may each and all be made efficient substitutes for a long sitting. Our own Fothergill is again quoted as an example of unwearying pleasant kindness to the poor. The great secret is to take an interest in the patient's case, and to exhibit an earnest desire to be useful. This manifested in the mode by which gentlemen are accustomed to display their feelings, will be sure to succeed in gaining the good will and confidence of the patient. A pleasant, soothing, kindly manner may thus, from day to day, bring solace to those labouring under even the most incurable diseases. Of course M. Simon has no sympathy for the rough-spoken imitators of Abernethy or Dupuytren. Firmness may be conjoined with gentleness; *medicus sit sermone potens;* persuasion should dwell on his lips.

Discretion is another quality necessary in medical practice. How many break the golden rule of not talking about their patients! These babblers go about from house to house—a long list in their hand—repeating the names and diseases of their patients with no other object than to gratify a prurient desire for scandal in themselves and their hearers. We scarcely know anything in worse taste than this sort of conduct. Secrets of families are thus often hinted at or improperly revealed, and the practitioner is a traitor to the interests of those who have confided in his judgment and discretion. It may be a duty, however, to reveal the secrets of a patient, as in a case of undetected criminals. This is a question on which much nice casuistry has been displayed, and the limits of which have not yet been defined. In fact, the practitioner must take his own conscience as a guide in the matter. For ourselves, we would denounce a murderer, but not a poacher.

A chapter is devoted to a consideration of the duties which the practitioner owes specially to the sex. For his own interests he will endeavour to secure the good opinion of the ladies, as well by his kindliness of heart as his modesty of deportment. M. Simon dwells on this subject with all the grace and courtesy to be expected from an accomplished French gentleman. Women look upon their doctor as their friend: they are zealous for his interests; they are enthusiastic in his praise. This warm feeling of friendship renders them a little, but most excusably, *exigeant :* submission with a good grace is a duty. Rarely are females medical sceptics; rarely do they scoff at the art or the science, like those strong, bullet-headed men, who revel in the enjoyment of physical strength, and believe not in medical skill, because they know not disease. M. Simon would not, however, encourage the whimsical, the capricious, or the lascivious ; the little revenges practised by a few spasmodic movements induced *apropos* are not to be ministered to, nor the thousand absurdities of ladies with weak nerves.

M. Simon thinks special regard should be had towards old men. They fear death ; they are cowards in suffering ; they are querulous, obstinate, imperious ; and they are, therefore, the more likely to induce a feeling of disgust which may lead to cruel neglect, or a thinly disguised routinism. In old men the love of life is so strong, that if they heard of a country where people never die, to that they would go (the bull is M. Simon's,) to end their days. Nothing is more easy than to flatter and gratify this love of life by proper attentions, not forgetting sage hints from time to time that man, and especially an old man, is mortal. The warnings his sufferings give should be cautiously re-echoed ; one word in conversation, a doubtful answer, or a reluctant opinion, will be quickly understood, and produce better effects than a course of the most orthodox sermons. It is a remarkable circumstance that those old people who talk most frequently of their age and infirmities think them of the least importance. They have usually been invalids through life ; have had much experience of physicians ; and have one or more favourite theories by which they explain away the most threatening symptoms. M. Simon refers to the sensual desires of old men, and the love, in particular, of sexual pleasures they display. Of course such desires should not be pandered to ; they are, we believe, in many cases morbid phenomena, and significant of disease in the nervous centres.

M. Simon professes the most generous and sublime views as to the conduct of the practitioner in times of pestilence. We must confess that we have no sympathy with that heroic self-devotion he would have the practitioner display. We know by experience that society has always been most unjust towards those who have laboured at the greatest risk of their own lives, and of the lives of those dear to them; and who have regarded no labour or expense during a fearful epidemic. Our experience of the cholera pestilence is too recent to doubt that it is equally just and politic that society pay the practitioner in proportion to the risk he incurs, and the labour he undergoes. It is sacrifice enough if he keeps his post in the hour of danger. And yet how often were the most arduous services to the public, during the prevalence of the cholera, rewarded with a stingy and empty vote of thanks! We should be glad to see an efficient organization of the profession, if it were only for this :—that it might compel society in troublous times to be *just*.

M. Simon advises that when a fatal epidemic disease breaks out, the practitioner should immediately declare it to be non-contagious. We should be sorry to sanction such a step; our English notions of truth forbid us to counsel a lie. It is always impolitic, too, to do evil that good may come. M. Simon further states, that as one attack of some epidemics affords immunity from a second, this principle in pathology should be made popularly known, and applied even to those diseases to which it is not applicable. Courage and hope, he argues, would thus be sustained, and selfishness defeated. This is again to counsel a falsehood, and such counsel, we repeat, is as abhorrent to our principles of moral rectitude as to our views of sound policy.

A chapter is devoted to the question of circumspection in the use of remedies. We have from time to time denounced that mode of treating diseases which has been absurdly enough termed heroic, but which more properly should be termed murderous. The conviction, however, of the dangers and destructive effects of such a method is now becoming more and more general. M. Simon, we need scarcely add, on this point, is in accord with ourselves. But too great circumspection may degenerate into timidity, and this be really as injurious as heroism. "Saltem non nocere" is a better maxim than "melius anceps quàm nullum remedium;" but a passive inertness must not be confounded with careful medication.

M. Simon defines medical esoterism to be that mysticism with which the practitioner performs his daily duties, and which he is compelled to adopt by the prejudices and ignorance of his patients. He deceives in pity for the weaknesses and timidity of mankind; he practises dissimulation that he may the more effectually administer relief. M. Simon thinks that he may, for these reasons, be a hierophant without blushing. A long chapter is devoted to esoteric medicine, and the first topic introduced is, what sort of conduct should be adopted in the management of patients with incurable disease. It is well known to experienced practitioners that few persons who so suffer are willing to abandon all hope, and as soon as the truth is announced to them by their attendant, they seek for remedy elsewhere, and often become the dupes and prey of unprincipled charlatans. Many practitioners think it their duty to tell the truth plainly and bluntly, regardless how they hurt the feelings of their patients, or sacrifice their own interests. We believe this is usually done from a highly honorable principle of action, although occasionally it is mere tyranny; but we question whether, even when done with the best motives, it is eminently wise.

We hold, in the first place, that no one has a right to pronounce a disease to be absolutely incurable, and, thereupon, to cease treatment. So long as the patient gives the practitioner his confidence, so long is it his duty to attempt every means for the cure that his reading, experience, or meditation may suggest; taking care that the patient be made no worse, nor his family nor fortune injured. It may be, and we believe it often is, that the patient knows the general opinion as to the incurability of the disease from which he suffers, and yet he hopes for a cure in his own case, because he has selected, as he believes, an eminently skilful, experienced, and acute practitioner, from whom much may reasonably be expected. Besides, just as men think all men mortal but themselves, so the incurables think all like cases incurable except their own. Now, as we do not remind men of their inevitable mortalness, so we ought not to remind incurables of their inevitable incurableness.

M. Simon criticises at length the opinions of certain writers (amongst whom is Gideon Harvey, physician to William III) who have advocated systematic deception and chicane in the practice of medicine. Harvey's book was a refined satire on the therapeutics of his day. He knew what *all* men of ordinary penetration now know, that the conflicting theories and modes of treatment adopted in the profession have nearly similar results; that Nature cures; and that the triumphant pæans of the pretender are raised for a victory not his own. The use of placebos has been legitimate from time immemorial. They are often necessary to save the patient from himself. The hypochondriac is morally insane, and must be treated by the soothing system so successful in other forms of mental derangement. M. Simon thinks that we ought to reason with patients of this kind, an observation, we may remark, rather indicative of the inexperienced practitioner—inexperienced at least in this class of cases. The fact is, we might as well reason with lunatics; hypochondriacs, however experienced in extravagant medication, never learn wisdom; actually knowing the evils that drugs inflict upon them, they persist in seeking and swallowing more. In all such cases a disinterested anxiety for the patient's good shonld be the rule for deciding what is right and proper.

We think we need not dwell on the subject of the next chapter—namely, the question what remedial means should be absolutely forbidden. No honorable practitioner will place his art at the service of the worn-out debauché, or minister to the criminal passions or fears of the seducer. It is to be regretted that venereal diseases, and diseases originating in excessive venery, or self-pollution, have fallen into the hands of excommunicate practitioners, and ignorant, unprincipled quacks, because we hold that the principle is bad which estimates the importance of diseases by their origin. All diseases whatever, originate either directly or indirectly, from our natural depravity. Their origin, it is true, may be a matter of taste, and it may be difficult to decide whether it be more honorable to treat the results of unbridled gluttony and drunkenness than of careless fornication; but there can be no doubt as to the principle. All questions of treatment and of hygiene, connected either directly or indirectly with the sexual system, can be dignified by the practitioner. His personal conduct and the dignity of his object will give them a real or a doubtful importance. Certainly all ribald jests should be avoided, as well as all prudery; an honest, manly, business-like firmness should be the rule of conduct: going straight to the object with few words, unless reproof be necessary. When we remember how extensive the relations of the sexual functions are, and how imperious the instinct, and the more imperious when the man is the most inexperienced, there must be a large mass of human suffering that the prudish practitioner never has the opportunity to alleviate. We believe the venereal and obscene quacks gather the richest harvest of any mediciners.

An interesting question arises in considering how far experiments in medication may be carried. M. Simon thinks only the few who are properly qualified by opportunities, previous studies, and experience, should undertake the difficult duty of filling up the chasms in therapeutics. Experimentation is unjustifiable (except in incurable cases) by the ordinary practitioner. He must be content to follow, and not aspire to lead. M. Simon seems to object strongly to the use of arsenical preparations. He would have their use absolutely forbidden. In this point we differ much from M. Simon. Given with due care and attention, much good may be done by them.

We pass rapidly over the remainder of the volume. A chapter on the use of intimidation in treatment and prophylaxis is followed by another on euthanasia, and the treatment of the dying; and this ends the second division of M. Simon's subject. The third division considers the duties of practitioners towards society. The fourth is occupied with the *rights* of the profession, and comprises medical organization, and civic status, privileges, immunities, and rewards. As the questions discussed belong to hygiene and medical polity, and are thus only incidentally a branch of medical ethics, we will here hold our pen. In conclusion, we may honestly say of M. Simon, as Sir Kenelm Digby observes in his critique of Sir Thomas Brown, "His wishes and aims, and what he pointeth out, speak him owner of a noble and generous heart."

---

### ART. X.

